# Tuning Interior Nanogaps of Double-shelled Au/Ag Nanoboxes for Surface-Enhanced Raman Scattering

**DOI:** 10.1038/srep08382

**Published:** 2015-02-11

**Authors:** Weiqing Zhang, Mohsen Rahmani, Wenxin Niu, Serge Ravaine, Minghui Hong, Xianmao Lu

**Affiliations:** 1 Department of Chemical and Biomolecular Engineering, National University of Singapore, Singapore 117585; 2 Department of Electrical and Computer Engineering, National University of Singapore, Singapore 117576; 3 Centre de Recherche Paul Pascal, CNRS, Université de Bordeaux, Pessac, France

## Abstract

Double-shelled Au/Ag hollow nanoboxes with precisely controlled interior nanogaps (1 to 16 nm) were synthesized for gap-tunable surface-enhanced Raman scattering (SERS). The double-shelled nanoboxes were prepared via a two-step galvanic replacement reaction approach using Ag nanocubes as the templates, while 4-aminothiolphenol (4-ATP) as SERS probe molecules were loaded between the two shells. More than 10-fold enhancement of SERS is observed from the double-shelled nanoboxes than Ag nanocubes. In addition, the SERS of the double-shelled nanoboxes increase significantly with the decrease of gap size, consistent with the theoretical prediction that smaller gap size induces larger localized electromagnetic enhancement.

Surface-enhanced Raman scattering (SERS) is a spectroscopic technique that provides significantly enhanced Raman signals from molecules absorbed on the surface of specific metals with nanoscale roughness^1^. It has been generally accepted that SERS enhancement is the outcome of both electromagnetic (EM) and chemical (CM) effects; while EM effect is typically a few orders of magnitude higher than that of CM^2^,^3^,^4^. To maximize the EM enhancement, considerable research attention has been directed to plasmonic nanostructures separated with nanoscale gaps^5^,^6^,^7^,^8^,^9^,^10^,^11^,^12^,^13^,^14^,^15^. This is because a nanogap (also known as “hot-spot”) formed between two or multiple nearly touching plasmonic nanostructures can give an intense localized electromagnetic field (E-field) that may allow the detection of Raman signals from even a single molecule^16^,^17^,^18^,^19^,^20^,^21^. To date, plasmonic nanostructures with nanogaps as SERS-active substrate can be obtained via either lithography techniques or colloidal methods. Lithography techniques, such as electron beam lithography (EBL) and on-wire lithography (OWL), are effective for the fabrication of plasmonic nanostructures with controllable gaps, but limited with high fabrication cost and complex procedures^9^,^21^,^22^,^23^,^24^,^25^,^26^,^27^. Colloidal strategies, which are based on induced aggregation of nanoparticles to form randomly distributed SERS hot-spots, are facile and low-cost. While the aggregation or self-assembly of colloidal nanoparticles may produce small gaps easily^28^,^29^,^30^,^31^,^32^,^33^, the resulting gap sizes are typically random and irreproducible, so as the measured SERS enhancement factors (EFs)^17^,^34^,^35^. To improve the reproducibility and stability of SERS signals, nanostructures with interior gaps have been reported recently. For example, Lim et al. synthesized Au nano-bridged core-shell particles with a 1-nm interior gap loaded with Raman dyes. In their method, DNA-modified Au nanoparticles were employed as the seeds for the growth of a shell on the surface of the nanoparticles. Their core-shell Au nanoparticles exhibited stable and reproducible SERS signals^14^. Halas et al. also reported a Au core-shell structure with an interstitial silica layer, namely nanomatryoshka, for fluorescence enhancement and photothermal cancer treatment^36^,^37^. In these reports, the effect of different gap sizes was not investigated. Most recently, Duan et al. synthesized spherical core-shell Au nanoparticles with Raman dyes trapped between the core and the shell separated by a polymer layer^38^. The gap-dependent SERS was measured and the core-shell structures were employed for the detection of cancer cells. However, the formation of a uniform coating of a polymer layer and hence controlled gap size can be difficult. In addition, the use of polymer to form the gap may limit the choice of Raman probe molecules that can be loaded between the Au core and shell.

Here, we report that via a simple galvanic replacement reaction (GRR) approach, double-shelled hollow nanoboxes with precisely tuned interior nanogaps from 1 to 16 nm can be obtained for SERS study. By trapping 4-ATP SERS probe molecules in the nanogaps, the change in SERS EF with gap size was investigated systematically. Figure 1 shows the preparation of the double-shelled hollow nanoboxes. Firstly, Ag nanocubes were employed as sacrificial templates to react with HAuCl_4_ solution to form single-shelled Au/Ag alloy nanoboxes. The single-shelled nanoboxes were then allowed to absorb 4-ATP molecules via incubation, followed by electroless plating of a thin layer of Ag. Afterwards, a second GRR was performed on the Ag-coated nanoboxes to form double-shelled nanoboxes. Within each double-shelled nanobox, the inner and outer shells are separated with a nanogap where the SERS probe molecules are loaded. By controlling the thickness of the Ag coated on the single-shelled nanoboxes as well as the amount of HAuCl_4_ solution added in the second GRR, the size of the nanogap could be precisely tuned from 1 to 16 nm. This structure offers an ideal platform for the study of gap size effect on the SERS signals for molecules sandwiched between two metallic shells. Since no polymer or DNA molecules are used to control the gap size, the SERS measurement is free of interference from these species. In addition, the surface plasmon resonance (SPR) of the double-shelled hollow nanoboxes can be tuned into the near infrared (IR) region, further extending the applications of such SERS substrates for bio-imaging and sensing.

## Results and Discussion

**Figure 2:**
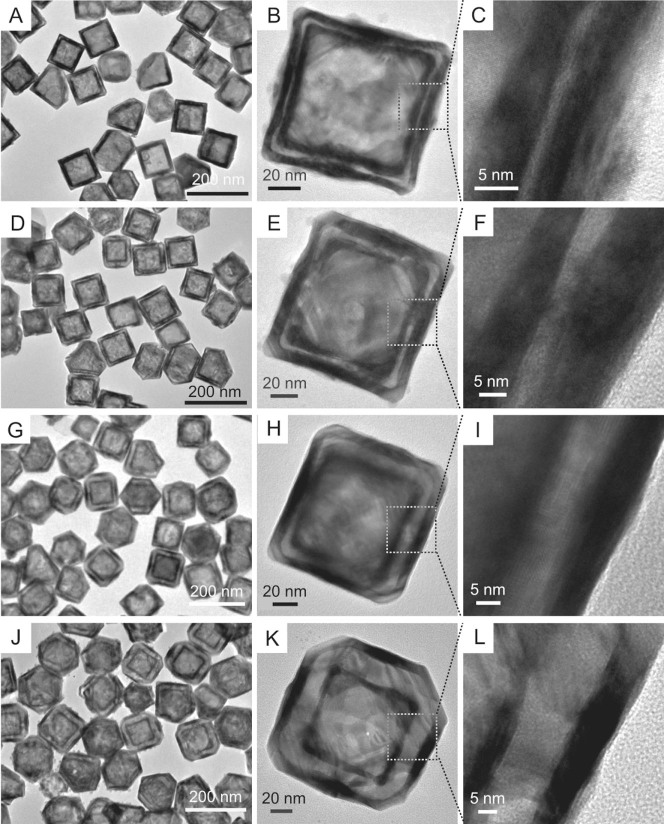
TEM images of the double-shelled Au/Ag nanoboxes with four gap sizes: (A–C) 1.2 nm, (D–F) 2.5 nm, (G–I) 8.0 nm, and (J–L) 15.6 nm.

Figure 2 shows the TEM images of the double-shelled nanoboxes with four interior gap sizes, namely 1.2, 2.5, 8.0, and 15.6 nm, respectively. As revealed from TEM images at low magnification (Figure S1), the double-shelled structures have relatively uniform sizes. In addition, the gap sizes of the double-shelled structures show small deviation. The control over gap sizes was realized by coating single-shelled nanoboxes with Ag of different thicknesses ranging from 4 to 21 nm (Figure S2). After the coating of Ag, the 4-ATP molecules adsorbed on the surface of each Au/Ag nanobox were buried underneath the Ag shell. Since 4-ATP has strong affinity to Au and Ag, these molecules can adsorb on the nanoboxes via Au-S and Ag-S bonds. In addition, the amino function group of 4-ATP can also bind to Ag, facilitating the growth of Ag layer during electroless plating on the surface of the nanoboxes^39^,^40^,^41^. Recently, Chen et al. reported that 4-mercaptobenzoic acid (MBA) as SERS probe molecules can be planted at the interface of Au@Ag core-shell structures^42^. The enhanced Raman signal in their study indicated that the probe molecules were located underneath the Ag shell. In the present work, to confirm the presence of 4-ATP molecules within the nanogap after the GRR with HAuCl_4_, EDX line scans were performed on individual double-shelled Au/Ag nanoboxes with different gap sizes. As shown in Figure S3, the EDX line profiles clearly show the signal of sulfur between the two shells, which can be only attributed to 4-ATP molecules adsorbed on both the inner and outer surfaces of outer and inner boxes, respectively. The presence of 4-ATP molecules on the inner surface of the outer box indicates that some 4-ATP molecules migrated inside the gap during the galvanic replacement reaction. In addition, although the Au-Ag ratios vary slightly for different gap sizes, they are all close to 1:1 (Table S1).

**Figure 3:**
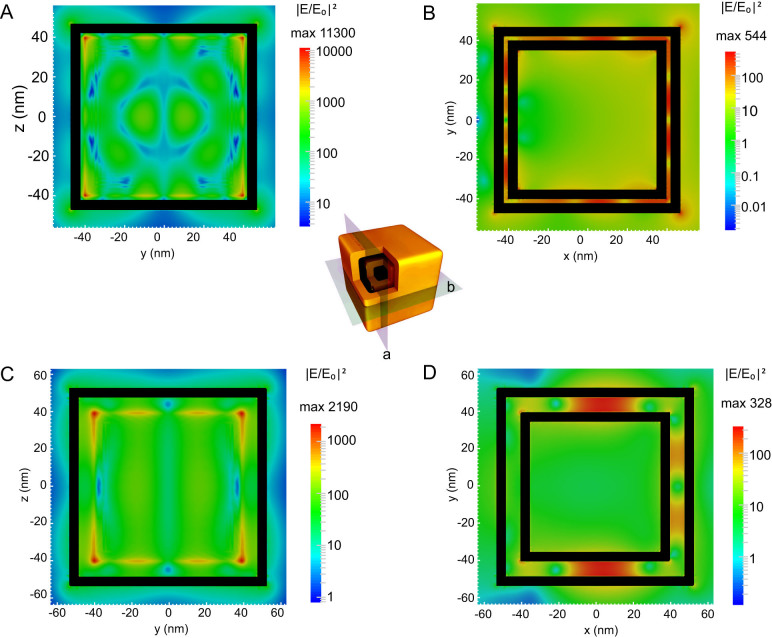
(A) 3D model of the double-shelled nanobox is shown in the center. Two cross-sectional planes are indicated - plane a located within the interior gap and coinciding with the outer surface of the inner shell; and plane b cutting through the center of the nanobox and parallel to one surface of the shell. The electromagnetic distributions of the two cross-sectional planes are calculated using DDA and presented in (A), (C) (for plane a) and (B), (D) (for plane b), respectively (black boxes represent the Au/Ag shells). The dimensions of the nanobox for DDA calculations are as follows: inner shell edge length = 40 nm; inner shell thickness = 5 nm; outer shell thickness = 5 nm; two gap sizes: (A–B) gap size = 2 nm; (C–D) gap size = 8 nm. The material used for the simulation is Au/Ag alloy with an atomic ratio of 1:1.

Discrete dipole approximation (DDA) calculation was conducted to compare the interactions of electromagnetic wave with the double-shelled nanoboxes of different interior gap sizes. For all calculations, the shell material consists of Au and Ag with an atomic ratio of 1:1 as estimated from EDX analysis (Figure S3, Table S1). Figure 3 shows the near-field electromagnetic field distributions of the double-shelled nanoboxes with gap sizes of 2 and 8 nm. For each gap size, two cross-sections of the nanobox are shown – one overlaps with one side surface of the inner shell (plane a), and the other one cuts through the center of the nanobox (plane b). Detailed analysis of the field distributions reveals three characteristics: i) the electromagnetic enhancement is highly localized within the interior gap, which is evident from the field distribution of plane b (Figure 3B, D); ii) the highest electromagnetic enhancement is located on the surface of the inner shell (plane a, Figure 3A, C), where the SERS probe molecules are adsorbed; iii) with the increase of gap size, the enhancement effect decreases -- as shown in Figure 3A, the maximum |E/E_0_|^2^ for 2-nm gap is larger than 10^4^, corresponding to an EF of 1.3 × 10^8^; while for 8-nm gap, the maximum |E/E_0_|^2^ drops to ~2000, corresponding to an EF of 4.8 × 10^6^ (Figure 3C).

**Figure 4:**
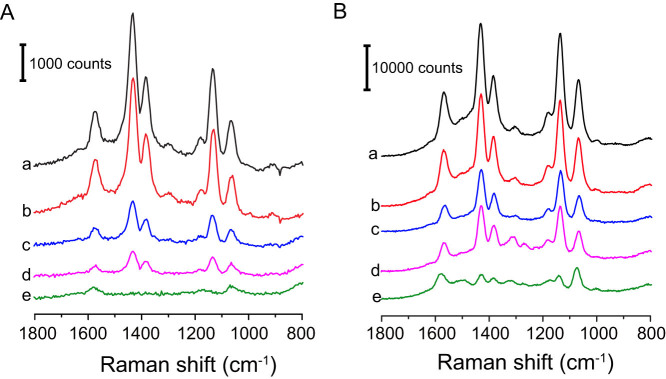
SERS spectra of 4-ATP with (A) 532-nm and (B) 633-nm lasers as excitation sources. For both excitation wavelengths: (a–d) are from 4-ATP molecules loaded in the interior gaps of double-shelled nanoboxes with gap sizes of (a) 1.2 nm, (b) 2.5 nm, (c) 8.0 nm, and (d) 15.6 nm; (e) is from 4-ATP molecules adsorbed on the surface of Ag nanocubes.

The SERS spectra of 4-ATP molecules loaded within the interior gaps of the double-shelled Au/Ag nanoboxes were recorded at excitation wavelengths of 532 (Figure 4A) and 633 nm (Figure 4B), respectively. The SERS spectra were obtained from colloidal samples with an acquisition time of 5 seconds. For all measurements, the concentration of nanoparticles was fixed at 4 × 10^10^ particles/mL, which was confirmed from the particle distribution profile obtained on Nanosight (Figure S4). As revealed from the SERS spectra, five strong peaks at 1070, 1135, 1385, 1433, and 1575 cm^−1^ can be observed from the double-shell samples. While the peaks at 1070 and 1575 cm^−1^ can be assigned to the a_1_ modes of 4-ATP molecules^30^,^31^,^43^, the assignments of peaks at 1135, 1385, and 1433 cm^−1^ are still under debating with two possible origins: i) b_2_ modes originated from charge transfer effect of 4-ATP^28^,^44^,^45^,^46^,^47^; or ii) a_g_ modes of p,p′-dimercaptoazobenzene (DMAB) formed from the dimerization of 4-ATP on metal surface^43^,^48^,^49^. It is well known that electromagnetic field (E-field) is strongly dependent on gap size^9^. As shown by curves a–d of Figures 4A and B, the intensity of each peak increases considerably as the gap size decreases. It should be noted that the number of SERS probe molecules is crucial to compare the SERS enhancement factor. Therefore, before Ag coating, samples with the same number of single-shelled nanoboxes and same concentration of 4-ATP were employed for the incubation. Assuming each 4-ATP has a footprint of 0.2 nm^2^ on the surface of Au/Ag nanoboxes with an edge length of 85 nm (Figure S2B)^50^, the number of 4-ATP molecules in the incubation solution is higher than required to cover the surface of the Au/Ag nanoboxes. Due to the strong Au-S and Ag-S bonds, it is reasonable to assume minimum loss of 4-ATP molecules from the nanoboxes during Ag coating and the following GRR. In addition, even if some 4-ATP molecules desorbed from the nanobox surface, it could only cause under-estimation of the SERS EFs^51^. Therefore, with the same particle concentration, the Raman signal of each sample should be from approximately the same number of 4-ATP molecules. As a result, the different spectral intensities can be translated into the change in SERS EFs of the nanoboxes with different gap sizes. Clearly, the double-shelled nanoboxes with smaller gap sizes exhibit much stronger enhancement effect. In addition, to compare the enhancement effect of the double-shelled nanoboxes with other nanostructures, the SERS spectra of 4-ATP molecules adsorbed on the surface of Ag nanocubes (70 nm) were also acquired. The corresponding spectra are shown as curves e in Figures 4A–B. For 532-nm excitation, when 4-ATP is adsorbed on the surface of Ag nanocubes, only weak a_1_ peaks at 1070 and 1575 cm^−1^ can be observed. Evidently, the double-shelled Au/Ag nanoboxes give much stronger SERS for 4-ATP relative to the Ag nanocubes. The same trend was also observed when the 633-nm laser was used as the excitation source.

**Figure 5:**
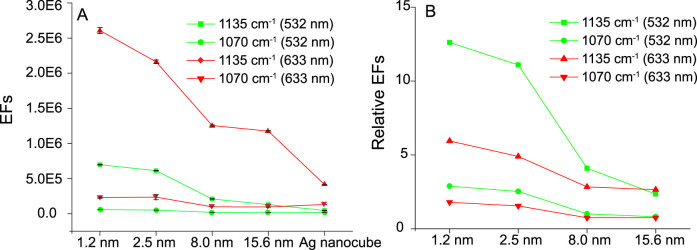
(A) EFs of Ag nanocubes and double-shelled Au/Ag nanoboxes; and (B) relative EFs (i.e., EF_double-shelled nanobox_/EF_Ag nanocube_) for peaks at 1135 and 1070 cm^−1^.

The SERS EFs for 4-ATP molecules in the interior nanogaps of the double-shelled nanoboxes were calculated. Peaks intensities at 1070 and 1135 cm^−1^ were selected for the calculation (detailed calculation can be found in Supporting Information). The resulting EFs were plotted in Figure 5A which clearly confirms that the EF of double-shelled nanoboxes increases significantly with the decrease of gap size. Specifically, for 1135 cm^−1^ mode at 532-nm excitation, the double-shelled nanoboxes with 1.2-nm gap show an EF of 6.6 × 10^5^, a 5-fold enhancement compared to that of 15.6-nm gap (1.2 × 10^5^). In addition to gap size, the decreased matching between the excitation wavelength and the SPR of the double-shelled nanoboxes with increased gap size may also contribute to the difference in EFs. The relatively EFs of the double-shelled nanoboxes were also obtained by comparing with that of Ag nanocubes (EF_double-shelled nanobox_/EF_Ag nanocube_, Figure 5B). Evidently, for all double-shelled nanoboxes, the EFs are much higher than that of Ag nanocubes, especially for nanoboxes with 1.2-nm gap at 532-nm excitation showing an EF that is 12 times higher than that of Ag nanocubes. These results clearly demonstrate that double-shelled nanoboxes with interior nanogap can generate strong electromagnetic field, leading to highly enhanced SERS signals for molecules loaded within the gaps.

In conclusion, double-shelled Au/Ag nanoboxes with precisely controlled interior gap sizes were obtained. More than 12-fold SERS enhancement was observed for 4-ATP molecules loaded in the nanogaps of double-shelled Au/Ag nanoboxes than those adsorbed on Ag nanocubes. In addition, the SERS EFs of double-shelled Au/Ag nanoboxes are strongly dependent on the gap size – with the decrease of gap size, the SERS EF increases significantly. The simple galvanic replacement reaction approach employed to control the interior gap size without the assistance of DNA, polymer, or silica may offer high flexibility in the selection of probe molecules and minimize the interference of other species with SERS measurements. The resulting double-shelled hollow nanoboxes with near infrared SPR can serve as an ideal platform for reproducible gap-tunable SERS and should be promising for applications in bio-imaging and sensing.

## Methods

### Synthesis double-shelled Au/Ag hollow nanoboxes

Silver nanocubes were synthesized with a modified polyol process^52^ and employed as the sacrificial templates to react with HAuCl_4_ solution. The resulting single-shelled Au/Ag alloy nanoboxes were then incubated with 4-ATP solution overnight and coated with a Ag layer via electroless plating. After the reaction with HAuCl_4_, double-shelled nanoboxes were obtained^53^. Experimental Details, DDA calculations, additional TEM images, UV-Vis spectra, EDX line profiles, the concentration profile of double-shelled Au/Ag nanoboxes, and the calculation of SERS enhancement factors can be found in Supporting Information.

### Characterizations

Transmission electron microscopy (TEM) images, high-resolution TEM (HRTEM) images, and energy dispersive X-ray spectroscopy (EDX) spectra were acquired using a JEOL JEM-2100F operating at 200 kV. All UV-vis spectra were recorded on a UV-1601 spectrophotometer (Shmadzu) at room temperature. The particle concentration of each sample was confirmed using Nanosight LM10-HS with a laser output of 60 mW at 405 nm. The Raman spectra were acquired from a Raman spectrometer equipped with a high sensitivity deep cooled CCD detector (Shamrock-163). Stabilized 532-nm and 633-nm laser diodes were used as the excitation light sources. The output powers of 532- and 633-nm lasers were fixed at 10 and 17 mW, respectively. The acquisition time of each spectrum was 5 seconds.

## Author Contributions

This project was carried out under the supervision of S.R., M.H. and X.L.; X.L. and W.Z. designed the experiments; W.Z. and M.R. performed the experiments; W.Z., W.N. and X.L. analyzed the results. All authors participated in manuscript preparation.

## Supplementary Material

Supplementary Information Supplementary information

## Figures and Tables

**Figure 1:**
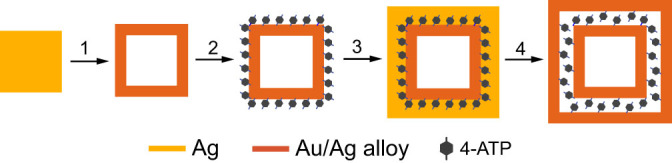
Preparation of double-shelled Au/Ag nanoboxes. Step 1, a single-shelled hollow nanobox formed via the GRR between a Ag nanocube and HAuCl_4_; Step 2, adsorption of 4-ATP molecules; Step 3, coating of a thin layer of Ag; Step 4, formation of double-shelled nanobox by reacting with HAuCl_4_.
