# Supplementary material for: Tuning Interior Nanogaps of Double-shelled Au/Ag Nanoboxes for Surface-Enhanced Raman Scattering
Source: Sci Rep. 2015 Feb 11;5:8382. doi: 10.1038/srep08382 (PMC4323660; doi:10.1038/srep08382)
Supplement: Supplementary Information [file srep08382-s1.pdf]

Supporting Information

Tuning Interior Nanogaps of Double-shelled Au/Ag Nanoboxes for Surface-Enhanced Raman Scattering

Weiqing Zhang,[1] *Mohsen Rahmani,*[2] *Wenxin Niu,*[1] *Serge Ravaine,*[3] *Minghui Hong,*[2,*] *Xianmao Lu* [1,*]

[1]Department of Chemical and Biomolecular Engineering, National University of Singapore, Singapore 117585
[2]Department of Electrical and Computer Engineering, National University of Singapore, Singapore 117576
[3]Centre de Recherche Paul Pascal, CNRS, Université de Bordeaux, Pessac, France

Emails: elehmh@nus.edu.sg; chelxm@nus.edu.sg

Materials and Methods

Chemical and Materials. Ethylene glycol (EG, J. T. Baker), silver nitrate ($AgNO_3$, Sigma-Aldrich), polyvinylpyrrolidone (PVP, Aldrich, Mw≈55,000), hydrochloric acid (HCl, Fisher, 37.5%), hexadecyltrimethylammonium bromide (CTAB, Sigma), sodium chloride (NaCl, Sigma-Aldrich), tetrachloroauric(III) acid ($HAuCl_4$, Aldrich), 4-aminothiophenol (ATP, Aldrich), L-ascorbic acid (AA, Sigma), and sodium hydroxide (NaOH, Sigma-Aldrich) were used as received. 18.2 MΩ•cm deionized water (DI-H_2O) was used in all experiments.

Characterizations. Transmission electron microscopy (TEM) images, high-resolution TEM (HRTEM) images, and energy dispersive X-ray spectroscopy (EDX) spectra were acquired using a JEOL JEM-2100F operating at 200 kV. All UV-vis spectra were recorded on a UV-1601 spectrophotometer (Shmadzu) at room temperature. The particle concentration of each sample was confirmed using Nanosight LM10-HS with a laser output of 60 mW at 405 nm. The Raman spectra were acquired from a Raman spectrometer equipped with a high sensitivity deep cooled CCD detector (Shamrock-163). Stabilized 532-nm and 633-nm laser diodes were used as the excitation light sources. The output powers of 532- and 633-nm lasers were fixed at 10 and 17 mW, respectively. The acquisition time of each spectrum was 5 seconds.

Synthesis of Ag nanocubes (NCs). Silver NCs (70 nm in edge length) were synthesized by a modified polyol process.[S1] In a typical synthesis, 10 ml EG was added into a 50 mL flask and heated under magnetic stirring at 140 °C for 1 hour. 2.1 mL of 3 mM HCl in EG was quickly injected into the flask using pipette. After 10 min, 6 mL of 94 mM $AgNO_3$ in EG and 6 mL of 147 mM PVP in EG were simultaneously injected into the reaction mixture using a two-channel syringe pump at a rate of 0.6 mL/min. This reaction mixture was allowed to react for around 24-36 hours at a temperature of 140 °C with constant stirring. Subsequently, the reaction was quenched by removing from the oil bath to an ice-water bath. The product was mixed with equal-volume acetone and centrifuged, following by washing with water for five times to remove excess PVP. The resultant NCs were dispersed in 20 mL of water in a vial that was wrapped with aluminum foil and stored in a dark cabinet for further use.

Synthesis of single-shelled Au/Ag hollow nanoboxes. Au/Ag hollow boxes were synthesized via galvanic replacement reaction (GRR).[S2] Typically, 0.5 mL aqueous dispersion of Ag nanocubes (the concentration of Ag was 13.8 mM based on ICP-MS measurement) was added to 14.5 mL of 20 mM PVP solution in a 50 mL flask. The flask was then heated in an oil bath at 100 °C for 10 minutes. 2.3 mL of 0.5 mM $HAuCl_4$ solution was injected into the reaction mixture using a syringe pump at a rate of 0.3 mL/min. The reaction mixture was continually stirred and heated at 100 °C for 10 mins before it was stopped and cooled down at room temperature. The solution was then transferred into a 20-mL vial.

Coating of Ag on single-shelled Au/Ag nanoboxes. 98.4 mg CTAB and 118 mg PVP were added to the solution containing Au/Ag nanoboxes obtained in the previous step. The final concentration of PVP and CTAB were 1 wt% and 18 mM, respectively. Afterwards, 0.6 mL of 10 mM 4-ATP in ethanol was added into the Au/Ag nanoboxes and incubated overnight. The solution (5 mL) was then heated at 70 °C for 20 minutes with stirring. To this solution, specific volumes of 4 mM $AgNO_3$, 0.1 M AA, and 0.1 M NaOH (volume ratio = 1:2:0.8) were added in sequence using pipettes. The resulting mixture was heated at 70 °C for 20 minutes under stirring. The product was then washed 3 times with DI-H_2O and 3 times with ethanol before it was re-dispersed in 2.55 mL of 20 mM PVP solution.

Synthesis double-shelled Au/Ag hollow nanoboxes. The aqueous dispersion of Ag-coated Au/Ag nanoboxes obtained in the previous step was transferred into a 20-mL flask, followed by adding 0.25 mL of 10 mM HCl. The mixture was heated with stirring at 100 °C for 5 min. 0.2 mM $HAuCl_4$ was then added to the mixture at a rate of 0.2 mL/min. The reaction mixture

was further heated for 5 min before it was cooled down to room temperature. The product was washed with saturated NaCl solution and DI-H_2O before it was re-dispersed in DI-H_2O.

DDA calculations. DDSCAT 7.2 was used for the calculation of the near-field electromagnetic field distributions of the double-shelled nanoboxes and Ag nanocubes with an incident wavelength of 633 nm.[s3] The incident electromagnetic wave (polarized along y-direction) propagates along x-axis. The complex refractive indices of the double-shelled nanoboxes for the simulation were determined based on a Au:Ag ratio of 1:1 (estimated from EDX analysis). Because the SERS spectra were obtained in solution, the interior gaps of the double-shelled nanoboxes were assumed to fill with water with a refractive index of 1.33.

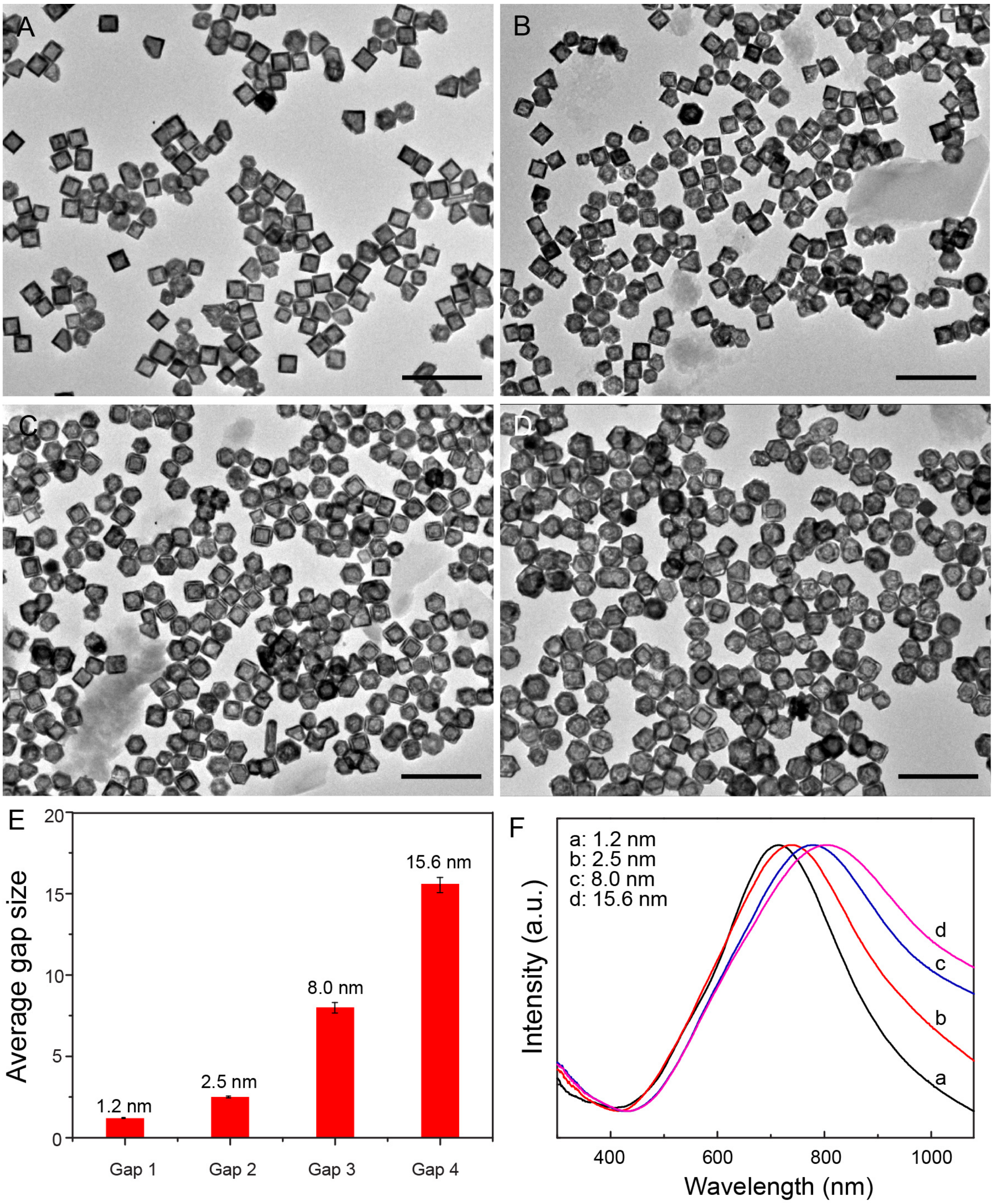

Figure S1. Low-magnification TEM images of double-shelled Au/Ag hollow nanoboxes with different gap sizes: (A) 1.2 ± 0., (B) 2.5 ± 0.1, (C) 8.0 ± 0.4, and (D) 15.6 ± 0.5 nm; (E) The measured average gap sizes for the four samples; (F) UV-vis extinction spectra – curve a for Ag nanocubes, and curves b-e for double-shelled Au/Ag nanoboxes with gap sizes of 1.2, 2.5, 8.0, and 15.6 nm, respectively. Scale bars are 500 nm.

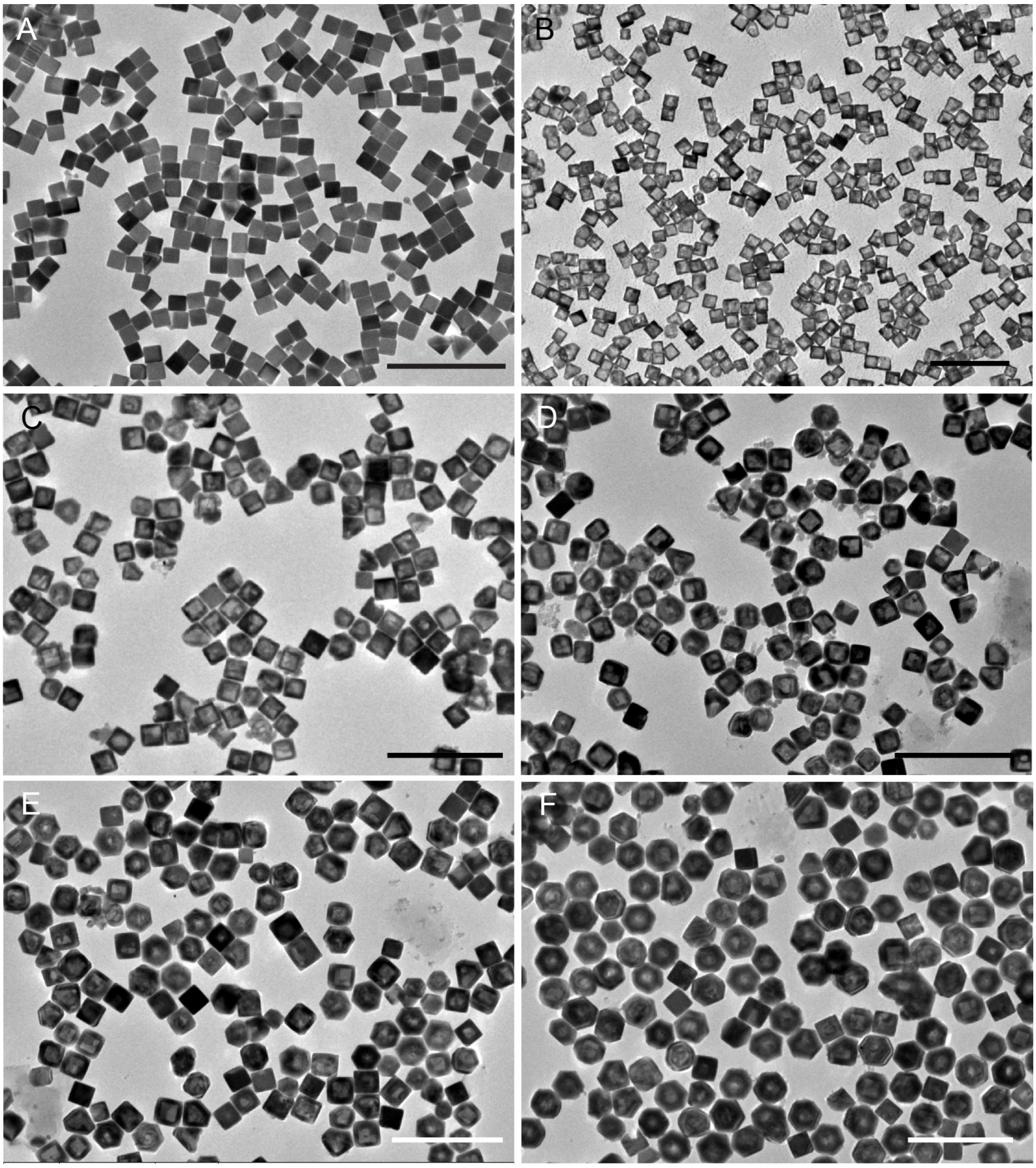

Figure S2. TEM images of (A) Ag nanocubes, (B) single-shelled Au/Ag nanoboxes, and single-shelled Au/Ag nanoboxes coated with Ag with thicknesses of (C) 4.3 nm, (D) 6.0 nm, (E) 11.0 nm, and (F) 21.1 nm Ag, respectively. Scale bars are 500 nm.

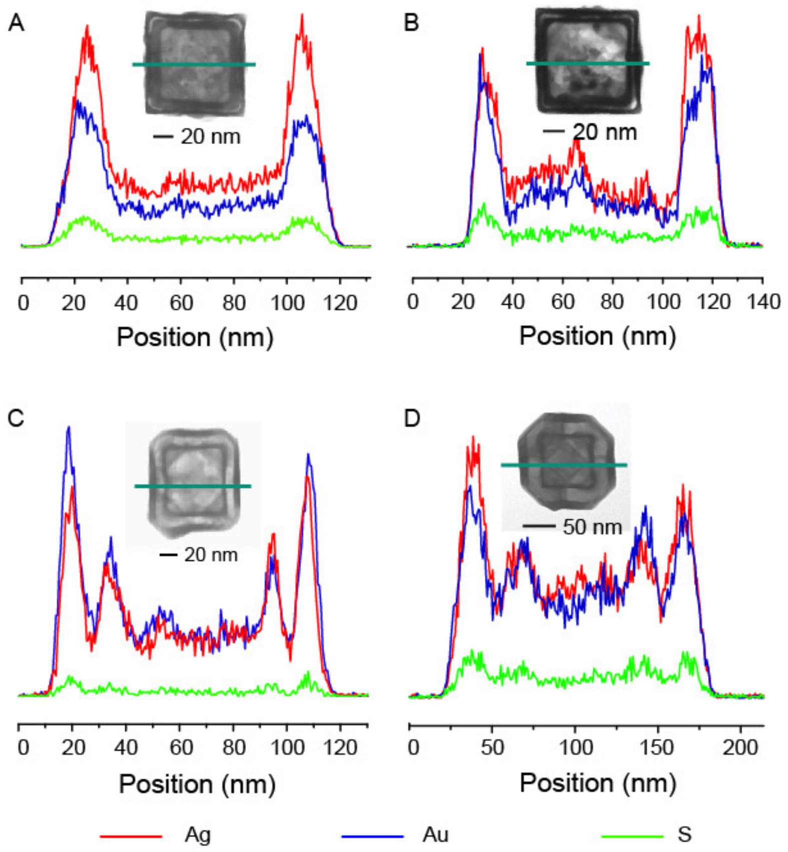

Figure S3. EDX line profiles of double-shelled Au/Ag hollow nanoboxes with different gap sizes: (A) 1.2 nm; (B) 2.5 nm; (C) 8.0 nm; and (D) 15.6 nm.

Table S1. Composition of double-shelled Au/Ag hollow nanoboxes with different gap sizes.

Gap size	Ag-to-Au atomic ratio
1.2 nm	1.35
2.5 nm	1.15
8.0 nm	0.82
15.6 nm	0.98

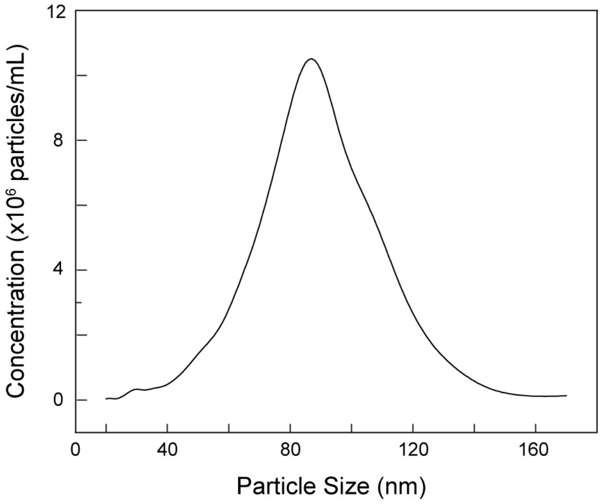

Figure S4. Representative particle concentration profile of double-shelled Au/Ag nanoboxes with 1.2-nm gap. The total particle concentration was obtained by integrating the curve over all sizes.

Calculation of SERS enhancement factors (EFs):

Peak intensities at 1073 and 1135 cm^{-1} were selected for the calculation of SERS EFs of double-shelled nanoboxes and Ag nanocubes. The ordinary Raman spectra were acquired with a 1.03 M 4-ATP solution in methanol with excitation wavelengths of 532 and 633 nm, respectively. The calculation is based on the following equation:

$$EF = (I_{SERS} \times C_{bulk})/(I_{bulk} \times C_{SERS})$$

Where I_{SERS} is the Raman peak intensity for double-shelled nanoboxes or Ag nanocubes, and I_{bulk} is for 4-ATP solution, C_{SERS} and C_{bulk} are the concentrations of 4-ATP loaded in double-shelled nanoboxes (or adsorbed on the Ag nanocubes) and in solution, respectively. C_{SERS} for double-shelled nanoboxes can be obtained as follows:

The total surface area of the Au/Ag nanobox (85-nm edge length of inner shell): $S_{nanoboxes}$ = 85 nm × 85 nm × 6 = 43350 nm^2;

Each 4-ATP molecule occupies an area of ~ 0.2 nm^2. Covering the whole surface of the inner shell requires $S_{nanoboxes} / (0.2 \times N_A) = 3.6 \times 10^{-19}$ mol of 4-ATP;

The concentration of 4-ATP for each sample of double-shelled nanoboxes (4×10^{10} particles/mL): $C_{SERS} = 3.6 \times 10^{-19} \times 4 \times 10^{10}$ mol/mL= 1.44×10^{-5} M.

REFERENCES

S1. Im, S. H.; Lee, Y. T.; Wiley, B.; Xia, Y., Large-Scale Synthesis of Silver Nanocubes: The Role of HCl in Promoting Cube Perfection and Monodispersity. *Angew. Chem. Int. Ed*. ***44***, 2154-2157 (2005).

S2. Sun, Y.; Xia, Y., Mechanistic Study on the Replacement Reaction between Silver Nanostructures and Chloroauric Acid in Aqueous Medium. *J. Am. Chem. Soc.* ***126*,** 3892-3901 (2004).

S3. Flatau, P. J.; Draine, B. T. *Opt. Express* ***20***, 1247-1252 (2012)
